# Phellinus linteus polysaccharides mediates acetaminophen-induced hepatotoxicity via activating AMPK/Nrf2 signaling pathways

**DOI:** 10.18632/aging.204260

**Published:** 2022-09-01

**Authors:** Lilei Zhao, Lianwen Zheng, Zheng Li, Meiyu Jin, Qi Wang, Jiaqi Cheng, Jinxia Li, Haihua Feng

**Affiliations:** 1 Key Laboratory of Zoonosis, Ministry of Education, College of Veterinary Medicine, Jilin University, Changchun 130062, Jilin, P.R. China; 2 Reproductive Medical Center, The Second Hospital of Jilin University, Changchun 130041, Jilin, P.R. China

**Keywords:** phellinus linteus polysaccharides, acute liver injury, APAP, Nrf2, AMPK

## Abstract

Overdose of acetaminophen (APAP) is currently one of the main causes of hepatoxicity and acute liver injury, which is often linked to oxidative stress. Phellinus linteus polysaccharides (Phps) have shown many hepatoprotective effects, however, the mechanism of Phps on APAP-induced acute liver injury has not been further elucidated. The aim of this study is to investigate the underlying mechanism of Phps to acute liver injury. The expression of AMPK/Nrf2 and autophagy were detected using western blot. The results indicated that Phps treatment effectively alleviated APAP-induced acute liver injury by reducing alanine transaminase (ALT) and aspartate aminotransferase (AST) levels in serum. Phps significantly attenuated myeloperoxidase (MPO) activity and glutathione (GSH) depletion. Meanwhile, Phps remarkably alleviated histopathological changes. Further research found that Phps promoted AMPK pathway and up-regulated nuclear factor erythroid-2-related factor (Nrf2) transported into nucleus, and elevated heme oxygenase 1(HO-1), glutamate-cysteine ligase catalytic (GCLC), glutamate cysteine ligase modifier (GCLM) and quinone oxidoreductase (NQO1). Additionally, Phps apparently facilitated the expression of autophagy proteins (ATG3, ATG5, ATG7, and ATG12). However, the protection of pathologic changes was nearly absent in Nrf2^-/-^ mice. Phps have potential in preventing oxidative stress in APAP-induced acute liver injury.

## INTRODUCTION

Drug-induced liver failure is one of the most important reasons resulting in liver disease in the world [1]. Acetaminophen (APAP) is a widely used antipyretic and analgesic, while, overused APAP can induce acute liver failure [2]. Studies have shown that APAP can be metabolized by hepatic cytochrome enzyme P450 (cytochrome P450, CYP450) into N-Acetyl-P-Benzoquinone Imine (NAPQI), which can bind to glutathione (GSH), to formation of APAP-glutathione, APAP-cysteine and APAP-N-acetylcysteine to detoxify [3]. However, excessive NAPQI will deplete intracellular GSH, and the remaining NAPQI covalently binds to cell biological macromolecules to form NAPQI protein adducts, disturbing the intracellular redox balance, and leading to oxidative stress injury, mitochondrial dysfunction, and nuclear DNA fragmentation, eventually, resulting in liver cell necrosis and liver damage [4].

Nuclear factor erythroid-2-related factor 2 (Nrf2) is a major antioxidant factor [5]. It has been reported that Nrf2 controls the expression of HO-1, GCLC, GCLM and NQO1 proteins [3]. In the event of oxidative stress, Nrf2 detaches from Keap1 and transfers into the nucleus and then combines with Maf. Nrf2-Maf heterodimer recognizes ARE to promote the expression of antioxidative and metabolic genes [6]. Nrf2^-/-^ mice are widely used to study the etiology and mechanism of many diseases [7–9]. Multiple studies in Nrf2^-/-^ mice have shown that Nrf2 has a protective effect on ethanol-induced hepatotoxicity [10]. AMPK makes sense of maintaining energy balance in cell [11]. Previous study reported that AMPK activation is implicated in several signaling pathways involved in nitric oxide production, sensing and responding to oxidative stress and inflammation [12]. AMPK also induced the antioxidative heme oxygenase (HO-1) gene expression through the Nrf2/ARE pathway [13].

Recently, studies have shown that autophagy is directly involved in the pathophysiology of APAP-induced liver injury [14]. Autophagy deficiency promoted ROS production, mitochondrial membrane depolarization, and JNK activation in APAP-induced liver injury [15]. Autophagy-related proteins ATG3, ATG5 and ATG7 have been reported to play an important role in promoting autophagy to improve liver injury [16–19]. APAP can form APAP adducts (APAP-AD) in mouse and human hepatocytes [20]. APAP-AD has also been detected in mitochondria and may contribute to APAP-induced mitochondrial dysfunction and subsequent oxidative stress [21, 22]. Autophagy can remove APAP-AD. Therefore, activated autophagy may have protective effect in APAP-induced acute liver injury.

Phellinus linteus polysaccharides (Phps) are extracted from *Phellinus linteus*, which is a well-known medicinal and edible fungus. Phps are the main active ingredient, mainly in the shape of polysaccharides, glycoproteins and glycosides [23]. Studies have shown that Phps have numerous pharmacological effects such as immune regulation, anti-tumor, and anti-oxidation effects, etc [24]. However, there is rarely study reported that the protective effect of Phps in mice acute liver injury. The purpose of this study was to investigate whether Phps could motivate the AMPK/Nrf2 pathway to protect APAP-induced acute liver injury, and to provide a theoretical basis for the development of hepatoprotective drugs.

## RESULTS

### Effects of Phps on AST, ALT, MPO and GSH in APAP-induced acute liver injury

**Figure 1:**
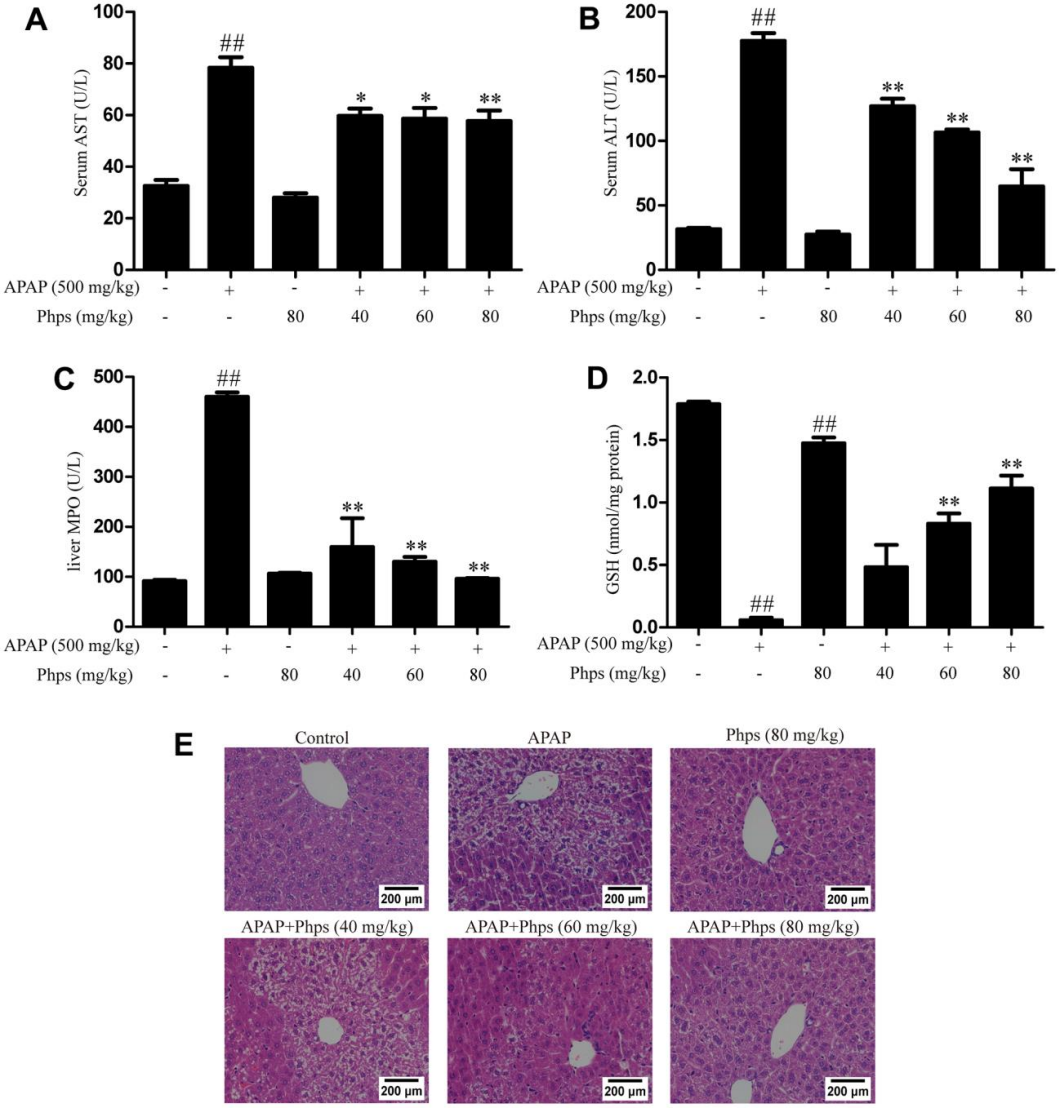
**Phps decreased the accumulation of AST, ALT and up-regulated the levels of GSH and down-regulated the levels of MPO in mice; Phps reduced the number of cell necrosis and the condition of structure damage of WT mice.** Haematoxylin and eosin staining of liver tissues. The mice were received Phps (40, 60, 80 mg/kg) prior 1 h APAP (500 mg/kg) injection. (**A**, **B**), the levels of AST and ALT. (**C**), the content of MPO in liver. (**D**), the levels of GSH in liver. (**E**) the liver sections by H&E staining (scale bars: 200 μm). All data are presented as mean ± SD (three independent experiments). ^#^*p* < 0.05 and ^##^*p* < 0.01 vs. control group; *p < 0.05 and **p < 0.01 vs. APAP group.

We tested the levels of AST and ALT in serum. Results indicated that the level of AST and ALT expressions were increased after treated with APAP (Figure 1A, 1B). While, wild-type mice treated with Phps showed obviously reduced compared with APAP group. GSH and MPO were necessary antioxidant in the detoxification of excessive APAP. The results showed that GSH was distinctly consumed (Figure 1D) and MPO were obviously increased (Figure 1C). The group treated with Phps could remarkably promote the level of GSH in liver and reduce the level of MPO.

### Effects of Phps on histological changes

The results of H&E staining showed that the hepatocyte structures were damaged and necrotic with APAP treated. The degree of liver injury was significantly reduced, the number of cell necrosis and the condition of structure damage were obviously improved (Figure 1E) after the treatment of Phps. These results suggest that Phps reduced APAP-induced acute liver injury.

### Effects of Phps on AMPK/Nrf2 pathway in APAP-induced acute liver injury

**Figure 2:**
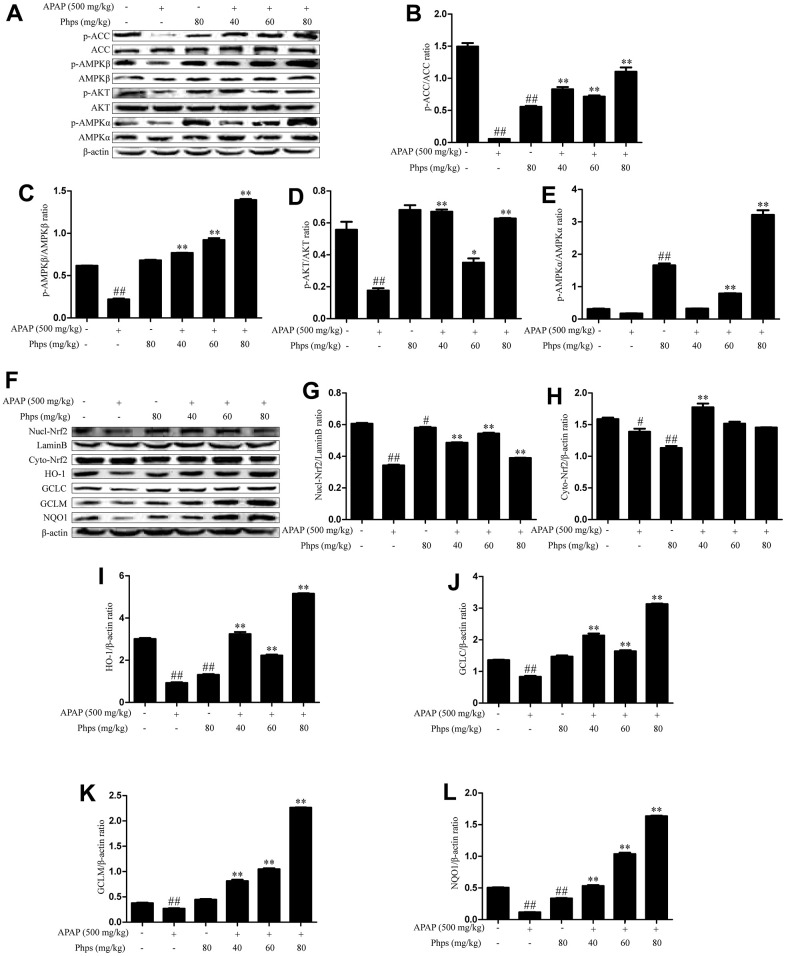
**Phps regulated the expression of p-ACC, p-AMPKβ, p-AKT, p- AMPKα, Nrf2, HO-1, GCLC, GCLM, NQO1 in APAP-induced acute liver injury of mice.** The mice were received Phps (40, 60, 80 mg/kg) prior 1h APAP (500 mg/kg) injection and liver was collected for Western blot. (**A**), the expression of p-ACC, p-AMPKβ, p-AMPKα and p-AKT in liver of mice. (**B**–**E**), Relative expression levels of all proteins including p-ACC, ACC, p-AMPKβ, AMPKβ, p-AKT AKT p-AMPKα and AMPKα. (**F**), the expression of Nrf2, HO-1 in nuclear protein and the expression of Nrf2, GCLC, GCLM, NQO1 in cytoplasmic protein. (**G**–**L**), Relative expression levels of all proteins including Nrf2, HO-1 in nuclear protein and Nrf2, GCLC, GCLM, NQO1 in cytoplasmic protein. All data are presented as mean ± SD (three independent experiments). ^#^*p* < 0.05 and ^##^*p* < 0.01 vs. control group; *p < 0.05 and **p < 0.01 vs. APAP group.

AMPK plays a key role in energy and inflammation reaction and affects nuclear transcription levels of Nrf2 [25]. Therefore, we tested the protein expression of AMPK pathway. As shown in Figure 2A, APAP significantly inhibited the expression of p-ACC, p-AMPKβ, p-AKT, and AMPKα. However, Phps distinctly increased ACC, AMPKβ, AKT, AMPKα phosphorylation levels (Figure 2B–2E). Nrf2 protects cells from oxidative stress by inducing the expression of protective genes. The levels of Nrf2 transfer into nucleus were significantly increased by Phps (Figure 2F). Meanwhile, the expression of NQO1, HO-1, GCLM and GCLC were promoted (Figure 2F).

### Effect of Phps on autophagy pathway in APAP-induced acute liver injury

**Figure 3:**
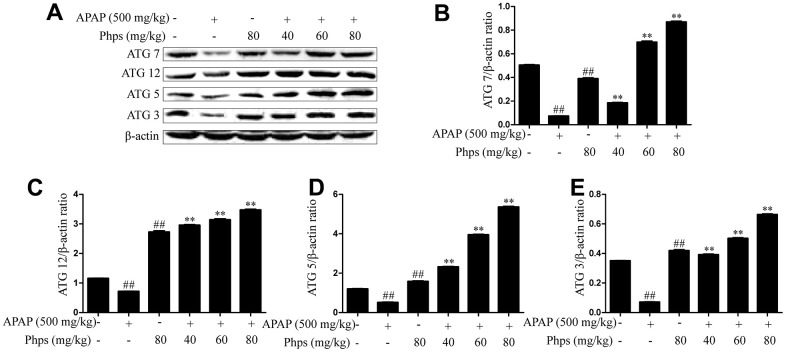
**Phps enhanced the expression of ATG7, ATG12, ATG5 and ATG3 in APAP-induced acute liver injury of mice.** The mice were received Phps (40, 60, 80 mg/kg) prior 1h APAP (500 mg/kg) injection and liver was collected for Western blot. (**A**), the expression of ATG7, ATG12, ATG5 and ATG3 in liver of mice. (**B**–**E**), Relative expression levels of all protein of all proteins including ATG7, ATG12, ATG5 and ATG3. All data are presented as mean ± SD (three independent experiments). ^#^*p* < 0.05 and ^##^*p* < 0.01 vs. control group; *p < 0.05 and **p < 0.01 vs. APAP group.

Autophagy can reply to oxidative stress, DNA damage and endoplasmic reticulum stress, which can participate in APAP-induced acute liver injury. The results shown in Figure 3 indicated that Phps could activate the expression of ATG7, ATG12, ATG5 and ATG3. Suggested that Phps could activate autophagy and increase the clearance capacity of the liver.

### Effect of Phps on APAP-induced acute liver injury were dependent on Nrf2

**Figure 4:**
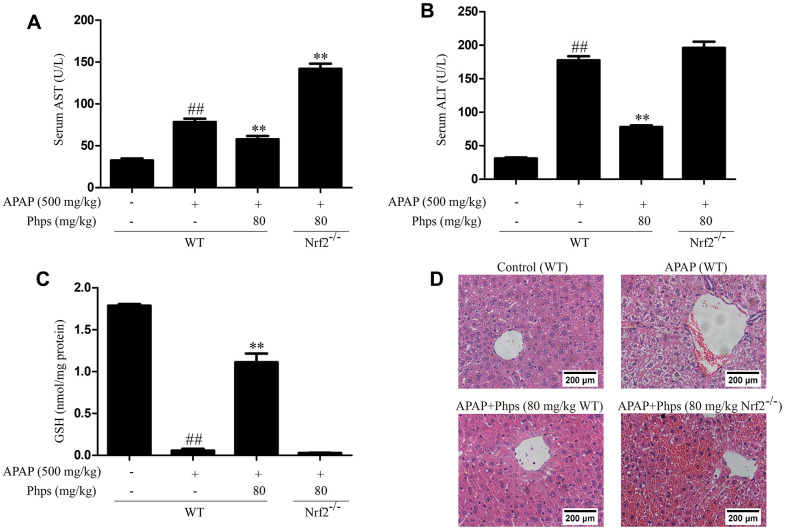
**Phps could not inhibit AST, ALT elevations and alleviate GSH consumption; Phps could not improve histopathological changes in Nrf2^-/-^ mice.** The mice were received Phps (80 mg/kg) prior 1 h APAP (500 mg/kg) injection (**A**, **B**), the levels of AST and ALT. (**C**), the levels of GSH in liver. (**D**) the liver sections by H&E staining (scale bars: 200 μm). All data are presented as mean ± SD (three independent experiments). ^#^*p* < 0.05 and ^##^*p* < 0.01 vs. control group; *p < 0.05 and **p < 0.01 vs. APAP group.

**Figure 5:**
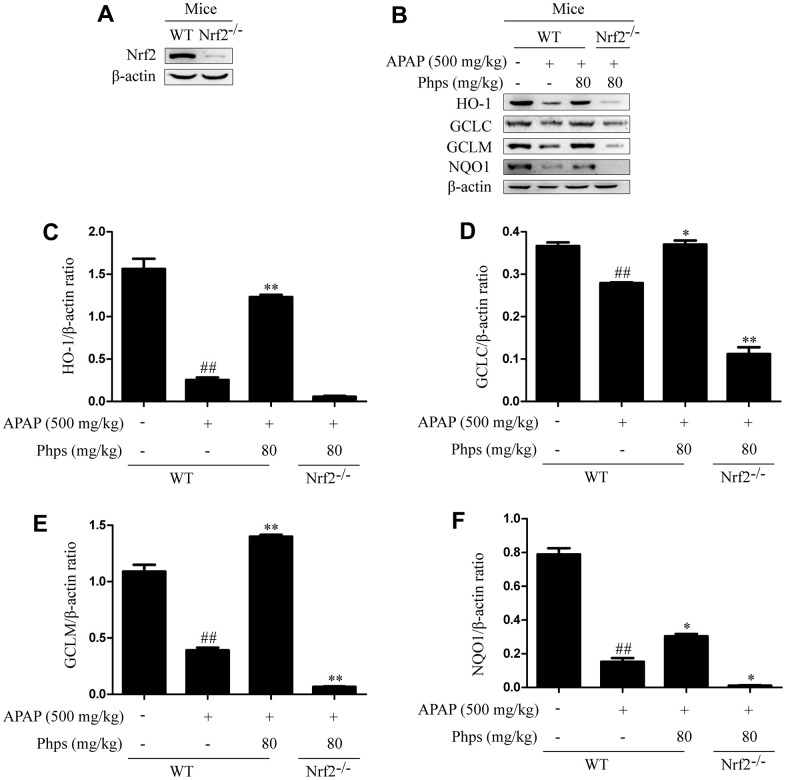
**Effect of Phps on APAP-induced acute liver injury were dependent on Nrf2.** The mice were received Phps (80 mg/kg) prior 1h APAP (500 mg/kg) injection and liver was collected for Western blot. (**A**, **B**) are protein bands of Nrf2 signaling pathway; (**C**–**F**) represent the ratio of HO-1, GCLC, GCLM and NQO1protein to β-actin. All data are presented as mean ± SD (three independent experiments). ^#^*p* < 0.05 and ^##^*p* < 0.01 vs. control group; *p < 0.05 and **p < 0.01 vs. APAP group.

In order to explore whether Phps alleviate acute liver injury through Nrf2 pathway, we used Nrf2^-/-^ mice in further study. Phps could not inhibit AST, ALT elevations and alleviate GSH consumption (Figure 4A–4C). We further examined histopathological changes and Nrf2 pathway proteins. As shown in Figures 4D, 5, with the Nrf2 knockout the protection of Phps were abolished in Nrf2^-/-^ mice.

## DISCUSSION

APAP-induced liver injury remains the major factor of acute liver injury in the world. It has been shown that metabolism, oxidative stress, autophagy, inflammation participated in the development of liver failure [26]. Overdose of APAP depletes GSH, leading to oxidative stress and dysfunction [27]. In our research, Phps pretreatment could obviously up-regulate express of GSH and down-regulate MPO levels. Therefore, we preliminarily concluded that Phps pretreatment can improve the level of liver antioxidant stress.

*Phellinus igniarius* is a kind of precious edible and medicinal fungus parasitic on mulberry plants. Phps is the main component of *Phellinus igniarius*, having high activity and antioxidant capacity. Our results shown that Phps has protective effect on APAP-induced acute liver injury. As well as Phps down-regulated the levels of AST and ALT in blood.

AMPK is found as a kind of energy sensor, but more and more studies have shown that AMPK plays an important role in energy and inflammation [28, 29]. AMPK also influences the level of Nrf2 nuclear transcription through GSK3β [30]. Nrf2 is an important part of antioxidative stress system. Nrf2 can protect body from oxidant stress and inflammation through adjusting a series of gene transcription (such as HO-1, NQO1). HO-1 has protective effect in cell and tissue damage, and has anti-inflammation and anti-oxidant [31]. Otherwise, GCLC and GCLM are participated in GSH synthesis steps, and the level of GSH reflects the ability of body anti-oxidant [32]. In this current study, Phps could improve AMPKα, AMPKβ and AKT phosphorylation, boosted levels of Nrf2 nuclear transcription as well. Moreover, the expression of anti-oxidant levels related proteins (GCLC, GCLM, HO-1 and NQO1) were significantly increased after protection by Phps, which improved the anti-oxidant ability of body. The results of Nrf2^-/-^ mice indicated that Phps has protective effect on APAP-induced acute liver injury through activated Nrf2 pathway.

Autophagy participates in multiple diseases. Study indicates that Autophagy was observed in hepatocytes and primary cultured cells of APAP-induced liver injury in mice [33]. Autophagosomes maintain cell homeostasis by degrading damaged organelles and biomolecules. The present study uncover that Phps could significantly up-regulate ATG7, ATG12, ATG5 and ATG3 levels, which is the same with previous studies [14, 34, 35].

In a word, our study demonstrated that Phps has a protective effect on APAP-induced acute liver injury in mice, and its main mechanism is to alleviate oxidative stress by activating Nrf2 signaling pathway; on the other hand, Phps activates autophagy pathway and relieves liver damage.

## CONCLUSIONS

In conclusion, as shown in the current study demonstrated that Phps treatments strongly protected APAP-induced acute liver injury against oxidative and autophagy. This research offers a piece of powerful evidence for the protective effects of Phps in APAP-induced acute liver injury.

## MATERIALS AND METHODS

Acetaminophen (APAP) was brought from Sigma-Aldrich (St. Louis, MO, USA), Phps was obtained from Baoyifeng Biology of Xi’an (Shaanxi, China). Glutathione (GSH), myeloperoxidase (MPO), aspartate aminotransferase (AST) and glutamic-pyruvic transaminase (ALT) detection kits were acquired through the Jiancheng Bioengineering Institute of Nanjing (Nanjing, Jiangsu, China). Primary antibodies against HO-1, AMPKα, phosphorylation-AMPKα (p-AMPKα), AMPKβ, phosphorylation-AMPKβ (p-AMPKβ), AKT and phosphorylation-AKT (p-AKT), were procured from Cell signal Technology (Boston, MA, USA). Antibodies against GCLC, GCLM, NQO1 were acquired by Abcam (Cambridgeshire, CA, UK). Anti-Nrf2 was gained from Affinity Biosciences. Antibodies against Lamin B, β-actin were acquired from Proteintech Group Inc. (Boston, MA, USA). HRP-conjugated goat anti-rabbit and goat anti-mouse antibodies were afforded by Boster Biological Technology (Wuhan, Hubei, China). All other chemicals were of reagent grade.

### Animals

Nrf2^-/-^ (knockout) and wild-type (WT) male C57BL/6 mice (18-22 g, 6-8 weeks old) were obtained by the Jackson Laboratory (Bar Harbor, ME, USA) and Liaoning Changsheng Biotechnology (Shen Yang, China). The mice were maintained in polypropylene cages in cages with the temperature of 24 ± 1° C (relative humidity 40-80%). All animal experiments were reviewed and approved by the Animal Welfare and Research Ethics Committee at Jilin University (approval number of ethics: SY202106005).

### Animals treatment

The wild-type mice were divided into 6 groups and treated as follow: control group, APAP (500 mg/kg) group, Phps (80 mg/kg) group, Phps (40 mg/kg) and APAP (500 mg/kg) group, Phps (60 mg/kg) and APAP (500 mg/kg) group, Phps (80 mg/kg) and APAP (500 mg/kg) group; the Nrf2^-/-^ mice were randomly separated into 4 groups: control group (WT), APAP (500 mg/kg) group (WT), Phps (80 mg/kg) and APAP (500 mg/kg) group (WT), Phps (80 mg/kg) and APAP (500 mg/kg) group (Nrf2^-/-^), 6 mice for each group. The mice were treated with intraperitoneal injection of 200 μL Physiological saline containing Phps (40, 60, 80 mg/kg). Injected APAP (500 mg/kg) 200 μL through intraperitoneal injection at 1h later. After 5 hours, the blood was collected through retro-orbital venous plexus. And then the liver tissues were removed for histopathological examination and protein extraction.

### Histopathological evaluation

The part of liver in each group was isolated and followed formalin fixation for 6 hours. All specimens were embedded by paraffin wax. And then we used hematoxylin and eosin (H&E) to evaluate the injury degree.

### Serum ALT and AST level

Bloods were placed at 4° C for 18 hours, and then centrifugated at 3000 rpm for 10 min. the serum were detected for ALT and AST followed with the kit instructions.

### MPO and GSH analyses

The liver tissues were homogenized with normal saline and then operated according to the instructions. The absorbancy was gauged at 460 nm. At last calculated the activity of MPO level followed by formula. The liver GSH level measured as the instructions. Briefly liver tissues were homogenized in special buffer solution at 4° C. Then, the absorbancy was detected at 412 nm. Finally, the liver GSH levels were normalized to the protein concentration.

### Western blot analysis

The liver tissues were lysed by Radio Immunoprecipitation Assay (RIPA) containing 1 mM phenyl methane sulfonyl fluoride (PMSF) to get protein. In addition, extracted nuclear and cytoplasmic protein was under the guidance of the instructions. The protein concentration was quantified using BCA protein assay kit, and equal amounts of protein (20 μg) were separated by 12% sodium dodecyl sulfate (SDS)-polyacrylamide gel. And then transfer onto polyvinylidene fluoride (PVDF) membranes. The membranes were blocked in 5% non-fat milk for 3 hours. Incubated with primary antibodies and second antibody. Subsequently the membranes were detected with enhanced chemiluminescence (ECL) and analyzed the bolt with Image-Pro Plus.

### Statistical analysis

Three independent data of experiment were collected and applied for one-way ANOVA by the GraphPad Prism 5.0. ^*^P<0.05 or ^**^P<0.01 make sense in statistics. The data was presented as mean ± SD. All the experiment results are repeated three times independently.

### Data availability statement

All relevant data about this research can be requested from the corresponding author.
